# Functional characterisation guides classification of novel *BAP1* germline variants

**DOI:** 10.1038/s41525-020-00157-6

**Published:** 2020-11-19

**Authors:** Jing Han Hong, Siao Ting Chong, Po-Hsien Lee, Jing Tan, Hong Lee Heng, Nur Diana Binte Ishak, Sock Hoai Chan, Bin Tean Teh, Joanne Ngeow

**Affiliations:** 1 Cancer and Stem Cell Biology Program, Duke-NUS Medical School, Singapore, 169857 Singapore; 2 Institute of Molecular and Cellular Biology, Agency for Science, Technology and Research, Singapore, 138673 Singapore; 3 Cancer Genetics Service, Division of Medical Oncology, National Cancer Center, Singapore, 169610 Singapore; 4 Cancer Science Institute of Singapore, National University of Singapore, Singapore, 117599 Singapore; 5 Genome Institute of Singapore, Agency for Science, Technology and Research, Singapore, 138672 Singapore; 6 Sun Yat-sen University Cancer Center, State Key Laboratory of Oncology in South China, Collaborative Innovation Center of Cancer Medicine, 510060 Guangzhou, Guangdong China; 7 Laboratory of Cancer Epigenome, Division of Medical Sciences, National Cancer Centre Singapore, Singapore, 169610 Singapore; 8 SingHealth/Duke-NUS Institute of Precision Medicine, National Heart Centre, Singapore, Singapore; 9 Lee Kong Chian School of Medicine, Nanyang Technological University, Singapore, 308232 Singapore; 10 Oncology Academic Clinical Program, Duke-NUS Medical School, Singapore, 169857 Singapore

**Keywords:** Clinical genetics, Genetics research

## Abstract

We have identified six patients harbouring distinct germline *BAP1* mutations. In this study, we functionally characterise known *BAP1* pathogenic and likely benign germline variants out of these six patients to aid in the evaluation and classification of unknown *BAP1* germline variants. We found that pathogenic germline variants tend to encode truncated proteins, show diminished expression of epithelial-mesenchymal transition (EMT) markers, are localised in the cytosol and have reduced deubiquitinase capabilities. We show that these functional assays are useful for *BAP1* variant curation and may be added in the American College of Medical Genetics and Genomics (ACMG) criteria for BAP1 variant classification. This will allow clinicians to distinguish between *BAP1* pathogenic and likely benign variants reliably and may aid to quickly benchmark newly identified *BAP1* germline variants. Classification of novel *BAP1* germline variants allows clinicians to inform predisposed patients and relevant family members regarding potential cancer risks, with appropriate clinical interventions implemented if required.

## Introduction

BAP1 (BRCA1-associated protein) is a nuclear localised deubiquitinating enzyme that is made up of a ubiquitin carboxyl hydrolase (UCH) domain, a host cell factor 1 (HCF1) binding domain, a C-terminal domain with a coiled-coil motif and a nuclear localisation signal^1–3^. BAP1 is known to be a tumour suppressor that is involved in many cellular processes including apoptosis, metabolism, DNA damage repair, regulation of gene transcription and removal of ubiquitin from histones (H2AK119ub)^4–10^. The polycomb group repressive deubiqutinase complex (PR-DUB) containing BAP1 and ASXL1/2 acts in opposition to polycomb repressive complexes (PRC)^2^. Together PRCs and PR-DUBs ubiquitinate and deubiquitinate histones, respectively, to fine-tune gene transcription in the cells. Loss-of-function mutations in *BAP1* result in loss of deubiquitination activities, disrupting various cellular processes including cell cycle, therefore driving tumourigenesis^1^ in many cancers such as renal cell carcinoma, mesothelioma, uveal melanoma, small cell and non-small-cell lung cancers and cholangiocarcinoma^11–14^.

*BAP1* germline variants predispose individuals to high risks of development of aggressive cancers, such as, uveal melanoma, mesothelioma, cutaneous melanoma and renal cell carcinoma at a younger age with poor prognosis^15^. Current therapeutic approaches for BAP1-deficient tumours are not particularly promising^16–20^. An increasing number of *BAP1* variants have been reported. For example, in 2018, 181 families have been discovered to carry a *BAP1* variant, of which 140 of the variants are unique^21^. Classification of missense *BAP1* variants is more difficult as compared to frameshift, nonsense and canonical splice variants even with in silico predictions^21^. As of April 2020, 466 *BAP1* *germline* variants of uncertain significance (VUS) have been reported on ClinVar, with 447 of them having single nucleotide variations and 404 of them resulting in missense mutations^22^. More of such *BAP1* variants are expected to be discovered as clinical laboratories have increasingly adopted next-generation sequencing to diagnose hereditary disorders due to its cost-effectiveness, high throughput and accuracy^23^. Given the increased risks and aggressiveness of cancer development and progression in patients with *BAP1* germline variants, understanding how variants in *BAP1* affect function will aid interpretation of uncertain variants. Classification of VUS allows us to inform predisposed patients and affected family members regarding potential cancer risks, with appropriate clinical interventions implemented if necessary.

Here, we study and characterise various *BAP1* germline variants identified in our patients. These patients have family histories of kidney cancers and other cancers such as breast cancer. Epidemiological, genetic and functional evidence are required to classify variants, and functional assays are ideal to elucidate pathogenicity status especially in rare variants^24^. To our knowledge, there are currently no recommended assays to reliably evaluate the functional impact of the germline mutations on BAP1 cellular function in the assessment of variant pathogenicity. Therefore, by using assays such as deubiquitination assays and immunofluorescence staining, we functionally compare *BAP1* germline VUS from our patient cohort against known pathogenic and likely benign variants, which may aid in classification of these variants.

## Results

### Clinicopathological characteristics of patients with germline *BAP1* variants

Through clinical genetic testing, we identified six patients harbouring germline *BAP1* mutations (Table 1, Supplementary Data 1). Three patients are carriers of pathogenic variants, two with variants of uncertain significance (VUS) and one with a likely benign variant. One of our patients had an additional VUS identified in *MSH6* (Supplementary Data 1). However, we prioritised the investigation of *BAP1* VUS in this study and the possible role of this *MSH6* VUS in tumorigenesis was not evaluated.

**Table 1:** Clinicopathological characteristics of our patients with *BAP1* germline variants.

Patient	BAP1 variants	Protein change	Clinical testing results	Gender	Cancer types and age of cancer diagnosis	Family history of cancers
S01053	c.852del	E284Dfs*51	Pathogenic	Female	Breast cancer (61) Thyroid cancer (65) Kidney ca. (66)	Aunt: Gastric cancer (85) Grandfather: Liver cancer (unknown age) Grandmother: Ovarian cancer (unknown age)
S01068	c.1358_1359del	K453Rfs*15	Pathogenic	Female	Breast cancer (36)	Mother: Breast cancer (52), Melanoma (56) Uncle: Gastric cancer (70) Uncle: Lung cancer (70) Uncle: Nose cancer (65) Cousin: unknown cancer (50)
S01093	c.588 G > A	W196*	Pathogenic	Male	Mesothelioma (53)	Father: Peritoneum mesothelioma (58) Aunt: Liver cancer (unknown age) Cousin: Liver cancer (unknown age)
S00199	c.551 A > G	D184G	VUS	Male	Kidney cancer (39)	Father: Kidney cancer (65) Grandmother: Breast cancer (65) Grandfather: Kidney cancer (65) Aunt: Ovarian cancer (47) Aunt: Kidney cancer (47)
S00676	c.299 T > C	L100P	VUS	Male	Kidney cancer (38)	Father: Nasopharynx cancer (65) Grandmother: Breast cancer (75) Aunt: unknown cancer (55) Uncle: Lung cancer (65)
S00723	c.1735G > A	G579R	Likely Benign	Male	Thymoma (39), Adrenal Cortical Carcinoma (48)	Sister: Breast cancer (48) Aunt: Breast cancer (unknown age) Aunt: Breast cancer (65)

**Fig. 1:**
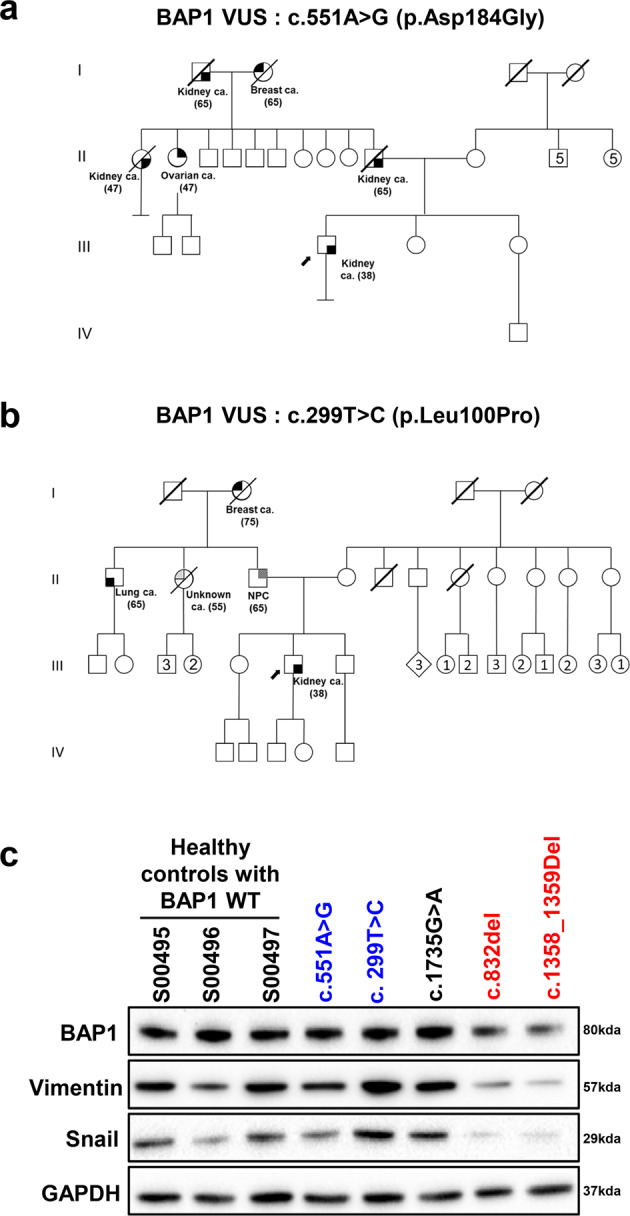
Pedigrees for patients with *BAP1* germline VUS and basal expression of EMT markers in patient-derived lymphoblastoid cells. Pedigrees for the cases identified with germline *BAP1* VUS c.551 A > G (p.Asp184Gly) (**a**) or c.299 T > C (p.Leu100Pro) (**b**). **c** Immunoblot using protein lysates extracted from patient-derived lymphoblastoid cells showed lower expression of full-length BAP1 in patient-derived lymphoblastoid cell lines with pathogenic *BAP1* germline mutations, accompanied by lower expression of Vimentin and Snail, representing reduced EMT processes. Variants in red or blue font represent pathogenic *BAP1* germline variant or *BAP1* germline VUS, respectively.

Among the pathogenic variant carriers, cancer spectrum was variable but consistent with *BAP1* tumor predisposition syndrome (Table 1): one presented mesothelioma, two with breast cancers, of whom one also presented multiple cancers including clear cell renal cell carcinoma (ccRCC). The median age at diagnosis is 39 (range: 36–61 years). Both VUS carriers were diagnosed with ccRCC in their late thirties, whereas the likely benign carrier presented thymoma and adrenal cortical cancer. All patients demonstrated strong family history of cancer (Table 1). Pedigrees for the cases identified with *BAP1* germline VUS show strong family history of cancers from the paternal side and early onset cancers, including renal and breast cancers (Fig. 1a, b).

### Patient-derived lymphoblastoid cell lines with *BAP1* pathogenic variants show reduced expression of full-length BAP1 and EMT markers

BAP1 is known to regulate migration, epithelial to mesenchymal transition (EMT) and therefore metastasis in various cancers including cervical cancer, breast, osteosarcoma and kidney^25–30^. Using patient-derived lymphoblastoid cell lines, we found that carriers of pathogenic *BAP1* germline variants (c.852_del and c.1358_1359del) had reduced expression of full-length BAP1, Vimentin and Snail, as compared to controls with wild-type BAP1 (BAP1 WT) (Fig. 1c), whereas *BAP1* germline VUS (c.299 T > C and c.551 A > G) or likely benign carriers were not significantly different from wild-type *BAP1* WT. Vimentin is a type III intermediate filament that drives EMT when overexpressed. Similarly, Snail also promotes EMT by repressing the expression of the adhesion protein E-cadherin. Loss of BAP1 has been reported to downregulate the expression of Snail, promoting ccRCC cells towards mesenchymal-epithelial transition^25^. Our results suggest compromised BAP1 function among the pathogenic variants carriers, which results in altered migration capabilities of the cancer cells, akin to the loss of BAP1.

### Effects of *BAP1* variants on cellular localisation

**Fig. 2:**
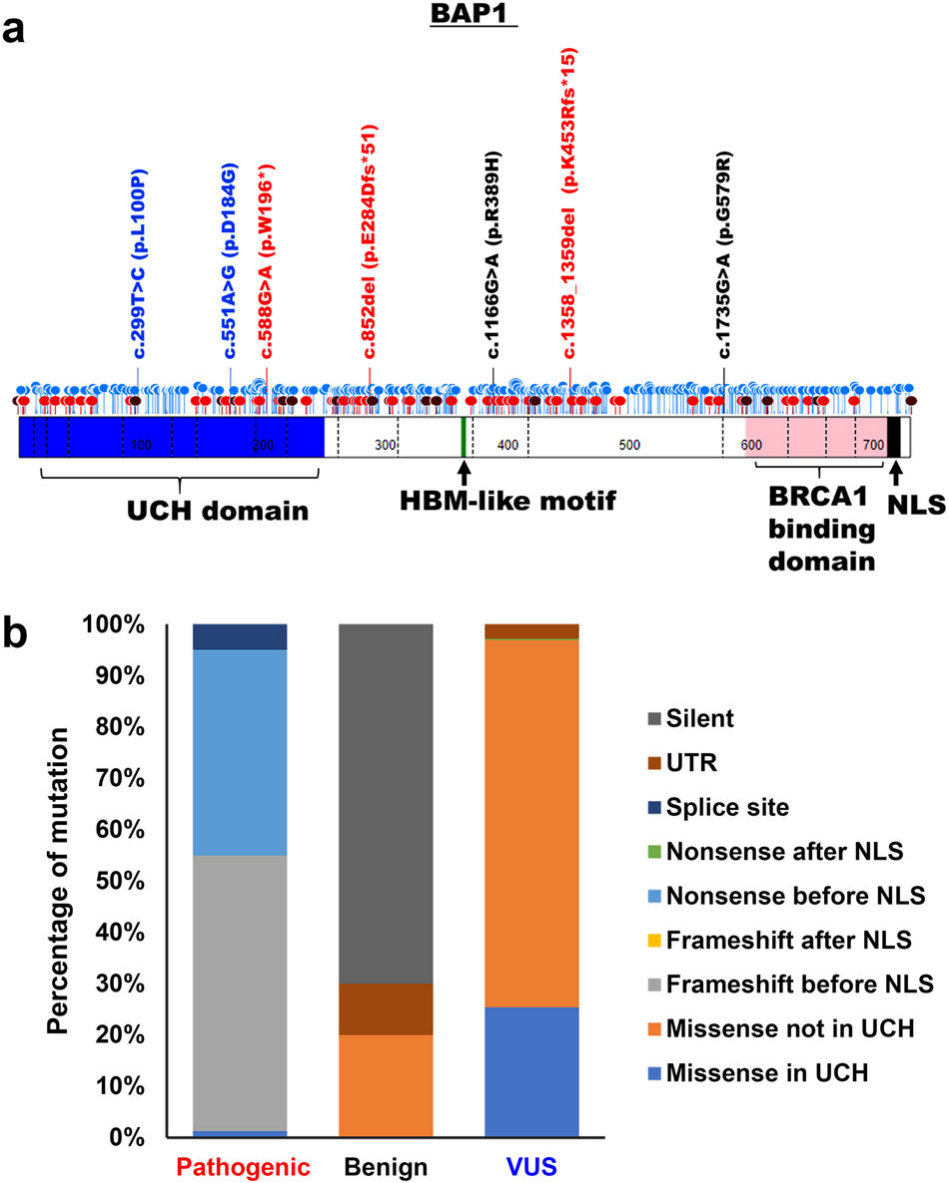
Schematic structure of BAP1. **a** Distribution of the *BAP1* germline variants. Positions of *BAP1* pathogenic variants, *BAP1* benign variants and *BAP1* VUS obtained from ClinVar are represented in red, black and blue lollipops, respectively. Our curated *BAP1* germline variants include one nonsense mutation (c.588 G > A: W196*), two frameshift mutations (c.852del: E284Dfs*51 and K453Rfs*15: c.1358_1359del) and four missense mutations (c.551 A > G: D184G, c.299 T > C: L100P, c.1166 G > A: R389H and c.1735G > A: G579R). Functional domains of BAP1 protein, consisting of UCH domain (1–250), HBM-like motif (363–366), interaction with BRCA1 (596–721) and nuclear localization signal (717–722). The figure was drawn using St. Jude PeCan Data Portal^57^. Variants in red or blue font represent pathogenic *BAP1* germline variant or *BAP1* germline VUS, respectively. **b** Percentage of the type of indicated mutation of *BAP1* pathogenic variants, *BAP1* benign variants and *BAP1* VUS based on information obtained from ClinVar.

Known mutation positions of *BAP1* pathogenic variants, *BAP1* benign variants and *BAP1* VUS are mapped as shown in Fig. 2a. *BAP1* pathogenic variants are mostly located before the nuclear localisation signal (NLS) and are predominately frameshift and nonsense mutations. Missense mutations observed in *BAP1* pathogenic variants are all found in the UCH domain of BAP1 (Fig. 2b). *BAP1* VUS appear to be distributed across the gene (Fig. 2a). While the majority of the missense mutations are not in the UCH domain of BAP1; 20–30% of such mutations are in the UCH domain of which function has not yet been established (Fig. 2b).

**Fig. 3:**
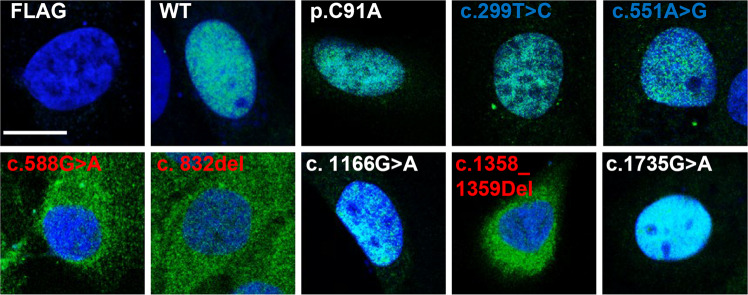
Effects of *BAP1* variants on cellular localization. Immunofluorescence staining using FLAG (green), nuclear stained with Hoechst 33342 (blue) to show localization of the BAP1 variants in HK-2 *BAP1* KO cells. Overlapping regions are cyan in colour. Pathogenic BAP1 germline variants showed cytoplasmic localization. Scale bar represents 15 µm.

To investigate if the genetic alterations lead to changes in the cellular localisation of BAP1, we expressed the different *BAP1* germline variants in HK-2 *BAP1* KO cell line. In addition to the variants identified in our patients, we also characterised the *BAP1* c.1166 G > A variant (p.R389H) that is not located in any known important domains of BAP1 (Fig. 2a) and *BAP1* c.271 T > G, c.272 G > C variant (p.C91A), a known mutation that abrogates the UCH function^31^. The nuclear localization of BAP1 is essential to its function, with the loss of nuclear localization observed in many cancers including uveal melanoma, mesothelioma and ccRCC^11,13,32–35^. *BAP1* variants that lead to protein truncation or frameshift before the nuclear localisation signal will change the localisation of BAP1, promoting oncogenic pathways^36,37^. Indeed, we observed that all three of our *BAP1* truncating variant carriers (c.588 G > A, c.852_del and c.1358_1359del) displayed cytoplasmic localisation of BAP1, which was not seen for the likely benign variant (c.1735G > A) or WT *BAP1* (Fig. 3), suggesting that the cytoplasmic localisation of BAP1 is consistent with the pathogenic classification for these variants. Of note, nuclear localisation of both *BAP1* VUS carriers (c.299 T > C and c.551 A > G) were unaffected (Fig. 3).

### Effect of gene mutations on deubiquitinase function

**Fig. 4:**
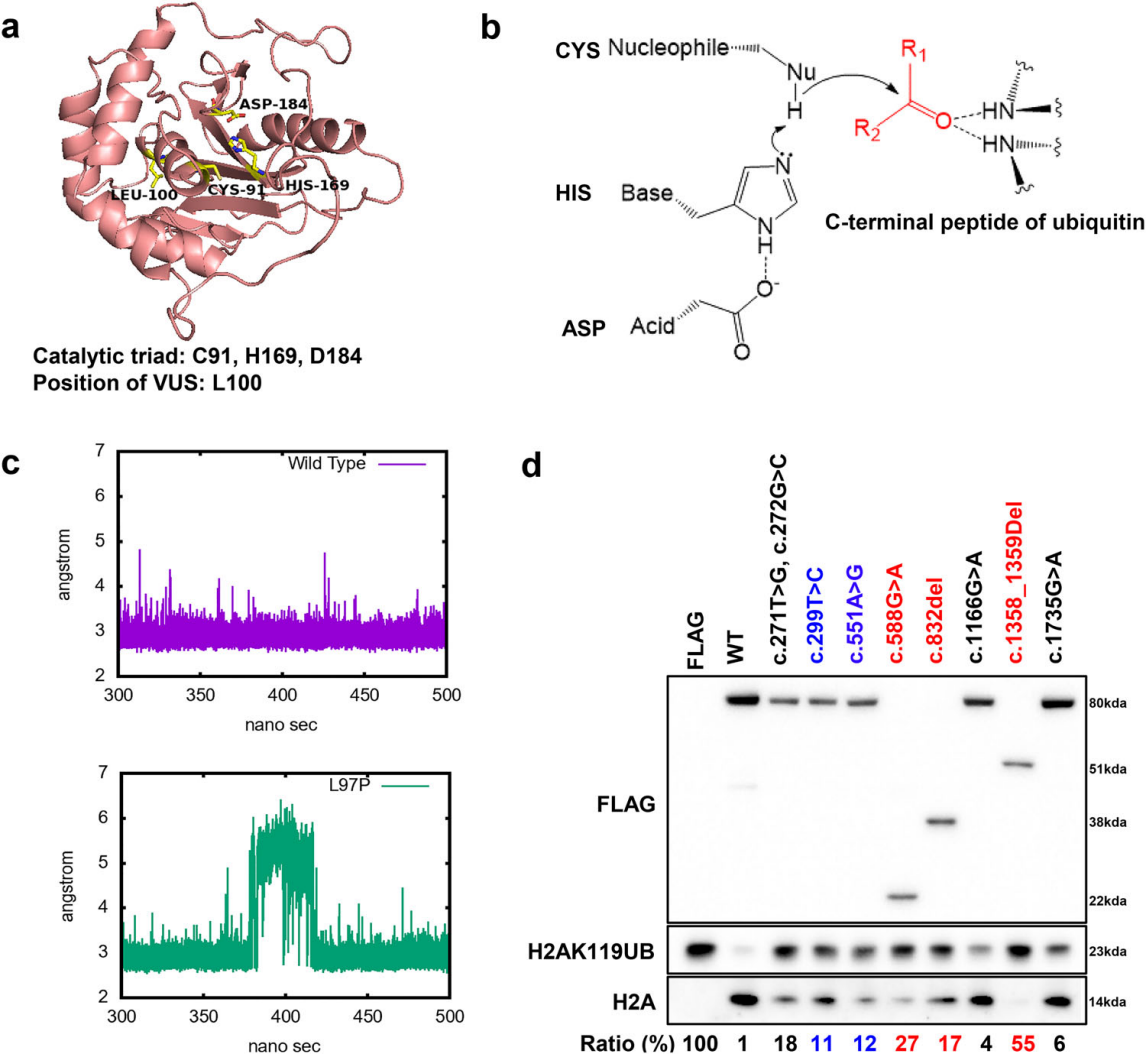
Effects of *BAP1* variants on deubiquitinase function. **a** Homology modeling of UCH domain using UCH-L5 (3ris.pdb) as a template to show the position of the catalytic triad (C91, H169, D184) in relation to the position of a VUS (L100) in the UCH domain of BAP1. **b** Deubiquitination reaction on a C-terminal ubiquitin tagged protein from the catalytic triad of UCH (made up of cysteine, histidine and aspartic acid). **c** The distance between the two residues of catalytic triad (Nε2 of H164 and Oδ of D179) of UCH-L5 during 300–500 ns of MD simulations. **d** In vitro deubiquitination assays using N-terminal FLAG tagged BAP1 expressed in HEK293T cells showed that the different BAP1 germline mutations affect the deubiquitination capability of BAP1. FLAG was used as a negative control and c.271 T > G, c.272 G > C (representing p.C91A mutation, known to lead to a catalytic dead domain) was used as a positive control. The ratio of intensity (in percentage) of H2AK119UB bands compared to H2A bands, normalized to FLAG expressing cell line, is shown below the immunoblot. The lower the ratio, the higher the deubiquitination activity of the variant. Variants in red or blue font represent pathogenic *BAP1* germline variant or *BAP1* germline VUS, respectively.

We performed homology modelling of the UCH domain of BAP1 using UCH-L5 (3ris.pdb) as a template (Fig. 4a). *BAP1* c.551 A > G (p.D184G) is in the catalytic triad of UCH (made up of cysteine, histidine and aspartic acid that abrogates its deubiquitinase function^38^. Deubiquitination by UCH domain takes place in two steps^39^ (Fig. 4b). The thiol of cysteine residue will first be deprotonated by histidine, followed by a nucleophilic attack by the deprotonated thiol on the carbonyl carbon of the substrate. The aspartic acid residue is likely important to keep the active site residues in a favourable geometry and to stabilise the ion pair formation of the catalytic residues^40^. All remaining *BAP1* variants are not located on the catalytic triad of the UCH domain, hence their effects on the deubiquitinase function of BAP1 protein are unknown (Fig. 4a). Our homology model of UCH domain of BAP1 using UCH-L5 (3ris.pdb) as a template shows that L100 of BAP1 is located close to the catalytic triad (Fig. 4a). To investigate the effects of the *BAP1* VUS p.L100P on the deubiquitinase function of BAP1 protein due to the conformational change of protein 3D structure, we would like to perform molecular dynamics (MD) simulations for BAP1 homology model. However, because of no structure of template covered, a long unstructured loop (10 amino acid) was modelled for Q156-M165. This region is close to H169 of catalytic triad and may affect the dynamic behaviour of catalytic triad. Instead, we performed MD simulations of its template/homologous protein, UCH-L5, for that the corresponding residue of L100 and adjacent residues of BAP1 is highly conserved among different species of BAP1 and human UCH-L5 (Supplementary Fig. 1). The corresponding position of L100 of BAP1 on UCH-L5 is L97 (Supplementary Fig. 1). We performed MD simulations for both wild-type and L97P of UCH-L5. The convergent and comparable Cα RMSDs over 500 ns simulations (Supplementary Fig. 2) implied that the structures of mutant UCH-L5 was as intact and stable as wild-type during simulations. However, during the last 200 ns stable simulations, the substantial conformational changes lasting ~30 ns were sampled on L97P mutant UCH-L5. The distance between the two members of catalytic triad (Nε2 of H164 and Oδ of D179) from L97P UCH-L5 was extended to >5 Å compared to the constant and stable ~3 Å in wild-type protein (Fig. 4c). The falling apart between catalytic triad may affect the enzymatic activity and implies that the highly conserved leucine (L100 of BAP1 and L97 of UCH-L5) may play an important structural role in supporting the spatial positions of catalytic triad of UCH domain.

In addition, we performed in vitro deubiquitination assay on H2AK119ub, a well-known substrate of BAP1, to assess the effect that the various mutations have on the deubiquitination function of BAP1 (Fig. 4d). *BAP1* p.C91A (c.271 T > G, c.272 G > C), a known amino acid mutation that abrogates the UCH function^31^ serves as our positive control. *BAP1* variants within the UCH domain, including the *BAP1* VUS p.L100P, displayed defective deubiquitination capabilities consistent with our positive control (Fig. 4d), suggesting defective UCH function. Interestingly, we found that the pathogenic *BAP1* germline variants also have impaired UCH function, even though not positioned within the UCH domain (Fig. 4d). By calculating the ratio of H2AK119ub band intensity compared to H2A band intensity, normalised to FLAG expressing sample, the pathogenic germline variants showed the greatest disruption to the deubiquitination activity of BAP1, while the likely benign variant showed the least disruption (Fig. 4d). Our results suggest that pathogenicity of the *BAP1* germline variant may be related to the integrity of the catalytic activities. Our *BAP1* germline VUS also showed impaired deubiquitinase function, albeit moderate compared to pathogenic *BAP1* germline variants (Fig. 4d).

### Functional characterisation of *BAP1* missense germline variants with conflicting interpretations

**Fig. 5:**
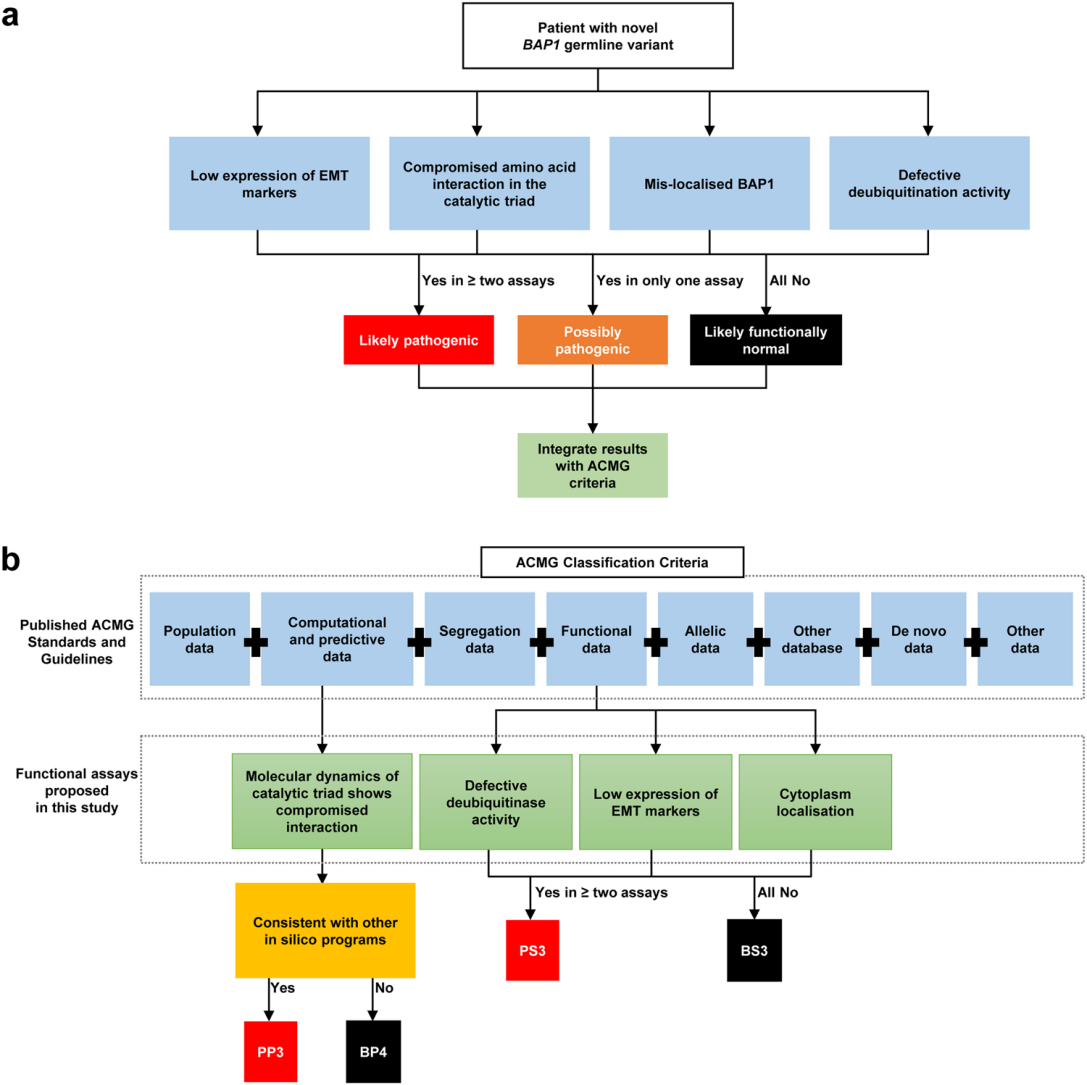
Summary of functional assays used in this study and proposed addition to the ACMG criteria to support *BAP1* germline variant classification. **a** Flowchart to access functionality of novel *BAP1* germline variant. Note that in silico molecular dynamics testing is used to determine compromised amino acid interaction in the catalytic triad. The rest of the assays are based on bench work in a laboratory. PDLCL refers to patient-derived lymphoblastoid cell line. If in agreement with other lines of evidence, variants with abnormality detected in one assay are possibly pathogenic and in two or more assays are likely pathogenic. **b** Proposed additional functional assays to support ACMG classification criteria for classifying *BAP1* germline variant. PP3 represents pathogenic supporting (in silico) BP4 represents benign supporting (in silico) and BS3 represents benign strong (functional studies) as per ACMG guidelines. PS3 also represents supporting level evidence in favour of pathogenicity (PS3_supporting) and BS3 also represents supporting level evidence in favour of benign interpretation (BS3_supporting) as per ClinGen recommendations.

Overall, we have functionally characterised *BAP1* germline VUS and compared them with known *BAP1* pathogenic and likely benign variants. In 2015, the American College of Medical Genetics and Genomics (ACMG) has published a standard framework containing variant curation criteria to guide assessment of variant pathogenicity^24^. The variants are assessed based on the strength of evidence of pathogenicity in these categories: population data, computational and predictive data, functional data, segregation data, allelic data, de novo data, other database and other data^24^ Subsequently, the Clinical Genome Resource (ClinGen) Sequence Variant Interpretation Working Group (SVI) proposed to refine criteria for variant interpretation from functional assays, recommending different levels of strength of pathogenicity to be assigned based on factors such as the type and number of controls used^41^. Based on results of the functional assays and number of controls used in the study, we have summarised our findings in a flowchart to aid in the determination of the functionality of a novel *BAP1* germline variant (Fig. 5a). These functional assays may be useful as supporting evidence for pathogenicity in the computational and predictive data and functional data categories of the ACMG criteria and supporting level evidence for pathogenicity according to the criteria proposed by ClinGen. Any functional abnormality detected using the functional assays described in this study will be considered evidence for pathogenicity as per ACMG guidelines; if in agreement with other evidence, variants with abnormality detected in one assay are possibly pathogenic and in two or more assays are likely pathogenic (Fig. 5b). Our results show that pathogenic variants tend to lead to the truncation of BAP1 and associated with reduced expression of full-length BAP1 and epithelial-mesenchymal transition (EMT) markers in patient-derived lymphoblastoid cell lines. Pathogenic *BAP1* variants also adversely affect deubiquitination capabilities and cellular localisation of BAP1. Combining the profiles of these assays will allow us to reliably distinguish between *BAP1* pathogenic and likely benign variants and therefore aid to benchmark newly identified *BAP1* germline variants.

**Fig. 6:**
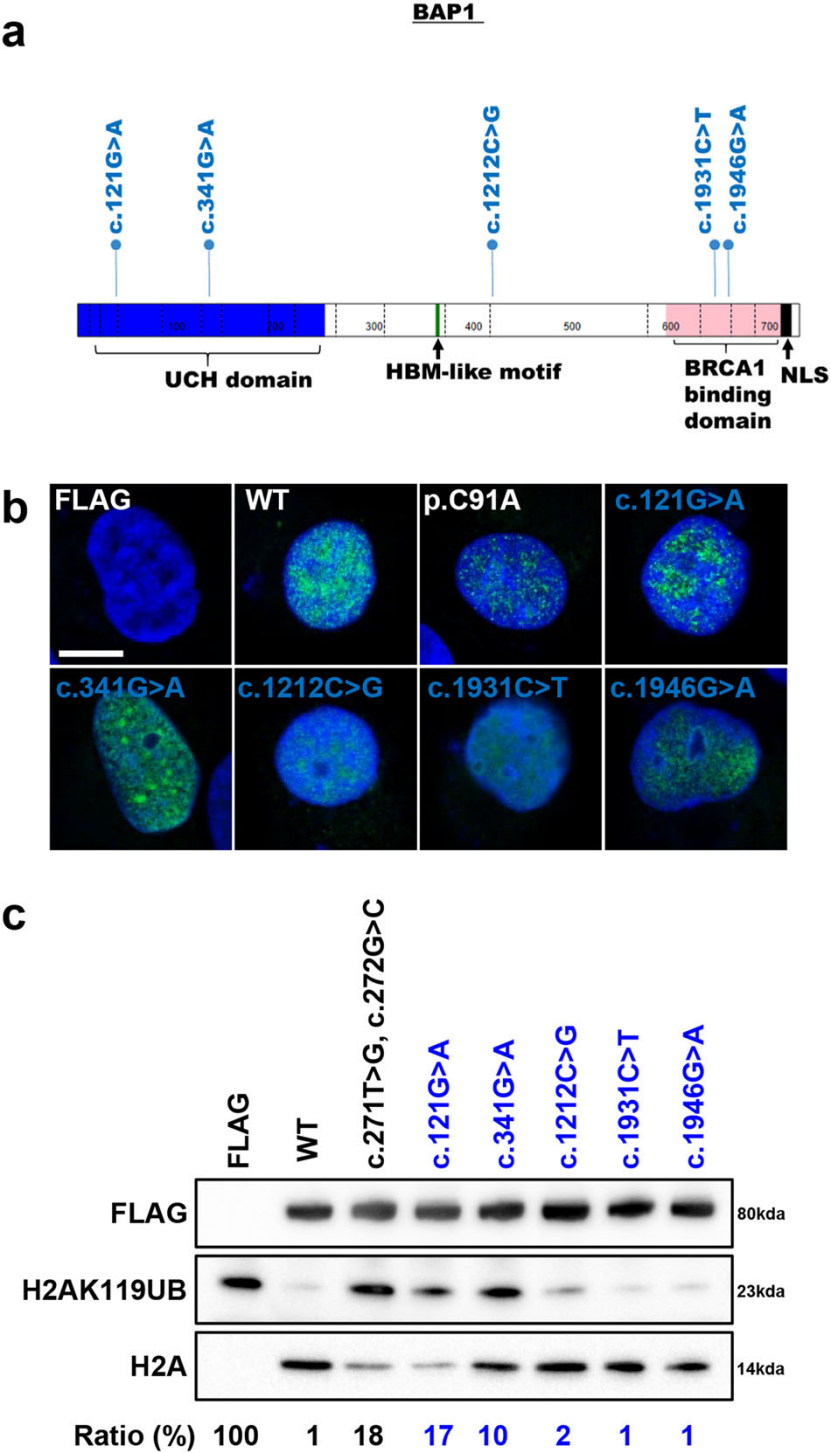
Functional characterisation of *BAP1* missense germline variants with conflicting interpretations. **a** Distribution of the *BAP1* missense germline variants with conflicting interpretations. Functional domains of BAP1 protein, consisting of UCH domain (1–250), HBM-like motif (363–366), interaction with BRCA1 (596–721) and nuclear localization signal (717–722). The figure was drawn using St. Jude PeCan Data Portal^57^. **b** Immunofluorescence staining using FLAG (green), nuclear stained with Hoechst 33342 (blue) to show localization of the *BAP1* variants in HK-2 *BAP1* KO cells. Overlapping regions are cyan in colour. Scale bar represents 15 µm. **c** In vitro deubiquitination assays using N-terminal FLAG tagged BAP1 expressed in HEK293T cells showed that the different *BAP1* germline mutations affect the deubiquitination capability of BAP1. FLAG was used as a negative control and c.271 T > G, c.272 G > C (representing p.C91A mutation, known to lead to a catalytic dead domain) was used as a positive control. The ratio of intensity (in percentage) of H2AK119UB bands compared to H2A bands, normalized to FLAG expressing cell line, is shown below the immunoblot. The lower the ratio, the higher the deubiquitination activity of the variant. Variants in blue font represent *BAP1* missense germline variants with conflicting interpretations.

17 *BAP1* missense germline variants with conflicting interpretations are listed on ClinVar as of July 2020. Five of these variants were picked for functional characterisation (Fig. 6a), to yield additional insights for these variants based on the proposed functional assays. Two of these variants are located in the UCH domain, two are located in the BRCA1 binding domain and one is located in region without any known domains (Fig. 6a). The nuclear localisation of these *BAP1* variants were not affected (Fig. 6b). In vitro deubiquitination assay showed that *BAP1* c.121 G > A and BAP1 c.341 G > A had impaired deubiquitinase functions, even though moderate compared to positive control (Fig. 6c). Our results provide functional evidence that *BAP1* c.121 G > A and *BAP1* c.341 G > A are possibly pathogenic variants and demonstrate that the proposed functional assays can be utilised to rapidly evaluate and facilitate classification of *BAP1* variants of uncertain significance to resolve conflicting interpretations for these variants.

## Discussion

*BAP1* tumour predisposition syndrome is a hereditary tumour syndrome that is associated with germline pathogenic mutations in *BAP1*^21^ and increased susceptibility for uveal melanoma, mesothelioma, cutaneous melanoma and renal cell carcinoma^42^. An increasing number of other cancers including cholangiocarcinoma, breast cancers and neuroendocrine tumours have also been shown to be associated with the syndrome^15,43–45^. The mode of inheritance in *BAP1* tumour predisposition syndrome is autosomal dominant and a majority of the germline pathogenic variants in *BAP1* are truncating^46^. Cancer patients with *BAP1* tumour predisposition syndrome are generally associated with poorer prognosis compared to patients without predisposition^15,43,45^. Germline carriers of *BAP1* pathogenic variants demonstrate earlier onset (e.g. median age of onset in uveal melanoma patients with germline *BAP1* mutations is 51 years compared to 62 years in the general population^47^) and more aggressive cancers^15^. Several therapeutic approaches have been explored for BAP1-deficient tumours, including chemotherapeutic drugs (e.g. gemcitabine), radiotherapy and small molecule inhibitors (e.g. EZH2 inhibitors) with limited success^16–20^. Due to the increasingly widespread use of next-generation sequencing in clinical laboratories^23^, more novel *BAP1* variants are expected to be discovered^21,22^. Functional studies have emerged as one of the indispensable methods to help classify *BAP1* VUS in addition to family cancer history and genetic evidence. Variant classification is important as it may have impact on subsequent cancer screening, follow-up and therapeutic strategies. Unfortunately, no standard protocol of functional assays is used to aid the classification of *BAP1* VUS.

In this study, we functionally assay each *BAP1* germline variant found in our patient cohort, including benign, pathogenic and VUS, using in vitro deubiquitination assay, cellular localisation determination using immunofluorescence staining and expression of EMT markers. These assays are only valid when the *BAP1* germline variant of interest can be expressed in cell models. By analysing known BAP1 pathogenic variants listed in ClinVar, we found that they are mostly located before the nuclear localisation signal (NLS) and are predominately frameshift and nonsense mutations (Fig. 2), which is consistent with that from our patient cohort. These types of mutations introduce a premature stop codon and are predicted to be target of degradation by nonsense-mediated mRNA decay (NMD) to protect the cells from the presence of abnormal peptides^48,49^. While we cannot exclude the possibility that our curated pathogenic frameshift and nonsense variants will undergo NMD, we have shown that truncated proteins can be expressed from these variants (Fig. 4d). With the exception of analysing the expression of EMT markers in the LCLs, our functional assays are based on introducing the *BAP1* germline variant into cell models and therefore cannot be used to investigate effects of loss of *BAP1* expression.

We found that pathogenic *BAP1* germline variants show defective deubiquitination function, are aberrantly localised in the cytosol or display low expression of EMT markers such as Vimentin and Snail, allowing them to be clearly distinguished from likely benign variants. Based on our deubiquitination assay, *BAP1* VUS c.299 T > C and c.551 A > T, both of which located in the UCH domain of BAP1, were observed to have decreased deubiquitinase activities. These patients also have strong family history of *BAP1*-related cancers. Therefore, *BAP1* VUS c.299 T > C (Supplementary Fig. 3a) and c.551 A > T (Supplementary Fig. 3b) are functionally abnormal. We have proposed the use of the functional assays used in the study to augment the ACMG criteria and ClinGen recommendations for assessing *BAP1* variant pathogenicity (Fig. 5). Given these lines of evidence and based on the level of evidence in the other categories in the ACMG framework, *BAP1* VUS c.299 T > C and c.551 A > T may therefore be considered to be reclassified as likely pathogenic (Table 2). Although our validation set of *BAP1* germline variants is limited in size, the profiles demonstrated by our functional assays could clearly distinguish the functional impact of pathogenic from benign variants and may be used to provide a line of functional evidence in determining the pathogenicity of newly identified *BAP1* variants.

**Table 2:** Proposed classification of two novel *BAP1* germline VUS using outcomes from additional functional assays used in this study.

*BAP1* variant	ClinVar/ dbSNP	Population Data (Allele Frequency)^a^	Computational and Predictive Data^b^	Functional Data^c^	Segregation Data	De novo Data	Allelic Data	Other Databases	Other Data	Proposed ACMG classification
c.551 A > G	VCV000412403.1/ rs1060503729	PM2 (not found)	PP3	PS3	NA	NA	NA	NA	NA	Likely pathogenic
c.299 T > C	NA/NA	PM2 (not found)	PP3	PS3	NA	NA	NA	NA	NA	Likely pathogenic

*NA* not applicable, *PM2* pathogenic moderate (allele frequency), *PP3* pathogenic supporting (in silico), *PS3* pathogenic strong (functional studies).

^a^Allele frequency was defined in reference to The Genome Aggregation Database (gnomAD).

^b^Computational and predictive data were based on outcomes from molecular dynamics stimulation used in this study and PolyPhen-2, PROVEAN, M-CAP and REVEL predictive in silico tools.

^c^Functional evidence was based on findings of this study.

In conclusion, our study may allow clinicians to quickly benchmark newly identified *BAP1* variants against known pathogenic and likely benign variants. Even though the penetrance of having a *BAP1* germline variant has been shown incomplete and the type of *BAP1-*related cancers has been observed to vary among different family members^42^, our findings contribute to providing functional support for resolution of *BAP1* variants with uncertain significance and facilitating clinical interpretation by clinicians so that appropriate prognosis, therapeutic intervention strategies and care may be delivered to patients with *BAP1* tumour predisposition syndrome.

## Methods

### Variant curation

Patients selected for this study were referred to our Cancer Genetics Service at National Cancer Centre Singapore for clinical genetic testing on multi-gene panels (including *BAP1)* by commercial laboratories accredited by CLIA/CAP. Classification of identified *BAP1* variants were performed by the respective accredited laboratories in accordance with the American College of Medical Genetics/Association for Molecular Pathology (ACMG/AMP) guidelines^24^. Patients included for analysis were identified to harbour germline *BAP1* variants, regardless of classification. Patient clinical history and family history of cancer were assessed by a clinical geneticist or genetic counsellor at our service. This study was approved by SingHealth Centralized Institutional Review Board (CIRB 2011/826/B) with signed informed consent from all participants.

### Commercial cell lines and cell culture

All commercial cell lines were purchased from ATCC. HEK293T is cultured in DMEM + 10% FBS. HK-2 *BAP1* KO cell is kind gift from Prof Teh Bin Tean’s lab and cultured in RPMI 1640 + 10% FBS. Patient-derived cell lines are cultured in RPMI 1640 + 10% FBS. All cell lines were maintained at 37 °C in a humidified chamber in the presence of 5% CO2 and routinely confirmed to be free of mycoplasma. Cells used for experiments were between 3 and 8 passages from thawing. At least three biological replicates were performed for each experiment.

### Immunoblotting

Cell pellets were collected and washed twice in cold PBS, before they are lysed in RIPA buffer supplemented with protease and phosphatase inhibitor cocktail (Roche). The lysed cells were then and sonicated for 10 cycles at 30 s on and 30 s off, high settings using a Biorupter (Diagenode) to ensure complete lysis. The lysates were clarified by centrifugation at 16,000 × *g* r.c.f for 20 min and the protein extracts were quantified using BCA assay (ThermoFisher Scientific Corporation, Waltham, MA, USA). 5–10 μg of protein was loaded onto 4–12% gradient gel, electrophoresed by SDS-PAGE and transferred to 0.2 μm nitrocellulose membrane (Biorad). The membranes were then blocked in 5% non-fat milk diluted in 1X PBST and incubated overnight with primary antibody diluted in blocking buffer at 4 °C. The following primary antibodies were used: Monoclonal Anti-Flag^®^ M2 antibody (Sigma–Aldrich: F1804, 1:2000), anti- ubiquityl-Histone H2A (Cell Signaling, Beverly, MA; #2577, 1:1000), anti-Histone H2A antibody (Abcam, Cambridge, MA, USA: ab18255, 1:1000), anti-BAP1 (Santa Cruz Biotechnology: sc-28383, 1;200), anti-Vimentin (Cell Signaling, Beverly, MA: #5741, 1:1000), anti-Snail (Cell Signaling, Beverly, MA: #3879, 1:1000) and GAPDH (Santa Cruz Biotechnology: sc-166545, 1;1000). Membranes were washed three times for 10 min each with 1× PBST then incubated with secondary HRP-conjugated antibody anti-rabbit or anti-mouse immunoglobulin (Promega Corporation: W4011 or W4021, 1:10,000) for 1 h. After washing three times for 10 min each with 1X PBST after incubation, the immunoreactivity was detected with SuperSignal West Femto Maximum Sensitivity Substrate (ThermoFisher Scientific Corporation, Waltham, MA, USA). All blots were derived from the same experiments and processed in parallel. Un-cropped images of blots are shown in Supplementary Fig. 4.

### Immunofluorescence staining

Transfected HK-2 BAP1 KO cells were plated on µ-Dish 35 mm imaging dishes (ibidi). Fixation was performed for 10 min using 4% paraformaldehyde (Sigma–Aldrich) at room temperature, followed by permeabilization using 0.3% Triton-X 100 (Sigma–Aldrich) on ice for 10 min. The cells were then blocked in 5% BSA in PBS for 1 h and then incubated overnight with Monoclonal Anti-Flag^®^ M2 antibody (Sigma–Aldrich: F1804, 1:500). Slides were washed three times with 1X PBS for 10 min and then incubated AlexaFluor A488 conjugated secondary antibodies (ThermoFisher Scientific Corporation, Waltham, MA, USA: A11008, 1:1000). Hoechst 33342 was used to stain the nucleus. After incubation for 1 h at room temperature, the slides were washed 3 times with PBS and mounted with Prolong Gold antifade reagent (ThermoFisher Scientific Corporation, Waltham, MA, USA). Images were acquired by confocal microscope (Zeiss LSM700).

### Homology modeling

Protein sequences of human BAP1 (Q92560) and human UCH-L5 (Q9Y5K5) were obtained from Uniprot^50^. The sequence identity of UCH domain between human BAP1 and UCH-L5 is 44%. The homology model of UCH domain of human BAP1 was built by MODELLER^51^ according to the template 3D structure of human UCH-L5 (3ris.pdb^52^).

### MD simulation

To investigate the effect of VUS on deubiquitination function due to conformational change of UCH domain, we implemented the computational approach, molecular dynamics (MD) simulation. The initial 3D structure of human UCH-L5 (3ris.pdb^52^ chainA, amino acids 7–227) was obtained from Protein Data Bank^53^. The structure of six missing residues (amino acid 155–160) were modeled by MODELLER^51^. Explicit hydrogen atoms and protonation states of titratable residues of UCH-L5 were determined by the web server PDB2PQR^54^ (http://nbcr-222.ucsd.edu/pdb2pqr_2.1.1/).

The systems of MD simulations were prepared using AmberTools package^55^. We adopted ff14SB force fields for the UCH-L5. The UCH-L5 was solvated in virtual cubic boxes filled with TIP3P water molecules. To maintain charge neutralization of the systems, proper numbers of counter ions (Na^+^ or Cl^−^) were added into the simulation boxes.

All energy minimizations and dynamics simulations in this study were performed using AMBER16 package^55^. To release steric clashes between atoms, we energy minimized the systems by the following protocol. The first 5000 cycles of energy minimization were performed by steepest descent method while the backbone heavy atoms of UCH-L5 were restrained by harmonic potentials (force constant was set as 10.0 kcal/mol-Å^2^). The second 5000 cycles of energy minimization were followed without any restraints.

Periodic boundary condition was employed during dynamics simulations while long range electrostatic interactions were treated by the particle mesh Ewald method and short range nonbonded cutoff was set as 12.0 Å. SHAKE algorithm was used to constrain bond length so that the simulations of production run allow a larger time step of 2 fs.

To heat up the system, we ran 100 ps simulations using time step of 1 fs. During the heating process, backbone heavy atoms of UCH-L5 were restrained by a harmonic potential with force constant of 10.0 kcal/mol-Å^2^. The dynamics simulations in this study were in the NPT ensemble. The pressure was coupled to 1.0 bar and temperature was coupled to 298.15 K by Berendsen’s coupling methods with both coupling constants of 1 ps. Backbone restraints were relaxed to 5.0 kcal/mol-Å^2^ for additional 100 ps simulation before production run.

During the simulations of production run, we performed 200 ns simulations for both systems with larger time step of 2 fs. The temperature coupling method of production simulations was switched to Langevin thermostat with the collision frequency as 5 ps^−1^ whereas Berendsen’s method was retained for pressure coupling. The simulations of production run were performed for 500 ns. All data analyses except heavy atom RMSD were based on the trajectories belonging to the period from 300 ns to 500 ns.

### In vitro deubiquitination assay

The indicated BAP1 variants were fused in frame with N-terminal FLAG in pFLAG-CMV1 expression vector (Sigma–Aldrich). HEK293T cells were transfected with 1 µg each of the indicated plasmids using Lipofectamine 3000 (ThermoFisher Scientific Corporation, Waltham, MA, USA). After 3 days, the cell pellets of the transfected cells were harvested, washed in PBS and lysed (50 mM Tris HCl, pH 7.4, 1% Triton X-100, with 150 mM NaCl). The FLAG tagged BAP1 variants were allowed to bind to Dynabeads™ Protein G (ThermoFisher Scientific Corporation, Waltham, MA, USA) that had been conjugated with Monoclonal Anti-Flag^®^ M2 antibody overnight. The FLAG tagged BAP1 variants bound to the beads were washed five times (0.5 M Tris HCl, pH 7.4, with 1.5 M NaCl). In vitro deubiquitination assay was performed as described^56^ with modifications. The washed beads with bound FLAG tagged BAP1 variants were resuspended in deubiquitination buffer (50 mM Tris-HCl, pH 7.3, 1 mM MgCl2, 50 mM NaCl, and 1 mM DTT, 1 mM EDTA and 1% Triton X-100). 200 ng of H2AK119UB (EpiCyher) was added to the mixture and deubiquitination was carried out at 37 °C for 3 h with gentle shaking. The reaction was quenched by adding 2× Laemmli buffer and analysed by immunoblotting.

### Reporting summary

Further information on research design is available in the Nature Research Reporting Summary linked to this article.

## Supplementary information

Supplementary Information

Supplementary Data 1

Reporting Summary

## Data Availability

All data generated or analysed during this study are included in this published article (and its supplementary information files).

## Code availability

Algorithms used for molecular dynamics stimulation detailed are in the Methods section.
