# Supplementary material for: Functional characterisation guides classification of novel BAP1 germline variants
Source: NPJ Genom Med. 2020 Nov 19;5:50. doi: 10.1038/s41525-020-00157-6 (PMC7678838; doi:10.1038/s41525-020-00157-6)
Supplement: Supplementary file 1 — Supplementary Information [file 41525_2020_157_MOESM1_ESM.pdf]

Supplement

1. Supplementary figures 1-4

2. Supplementary data 1 (separate excel sheet)

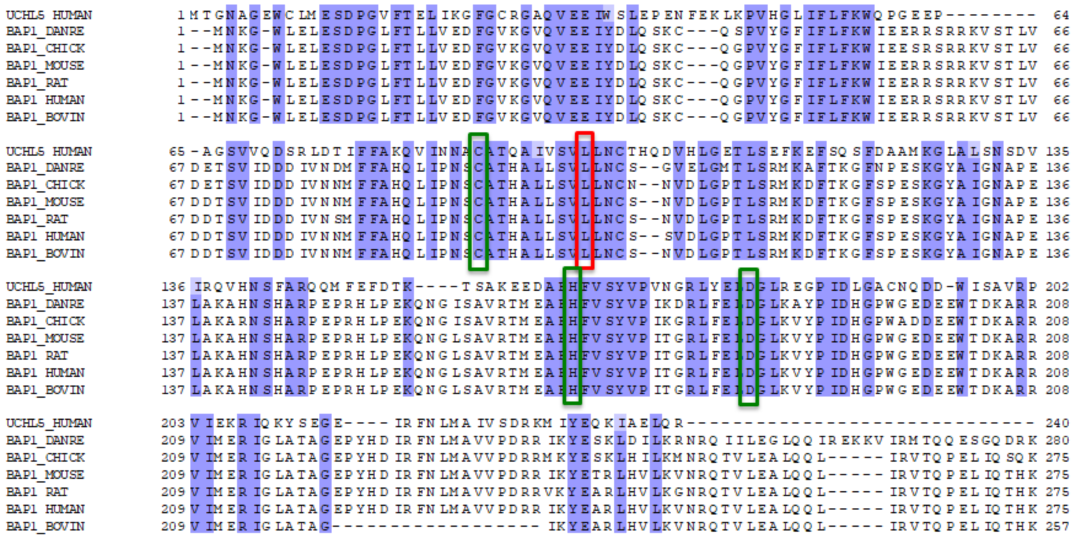

Supplementary Figure 1: Multiple sequence alignment of UCH domain among several species of BAP1 and human UCH-L5. Blue shadow marks the conserved residues. Green boxes mark the positions of catalytic triad. A red box indicates the position of conserved leucine which was identified as a VUS (L100P) of human BAP1.

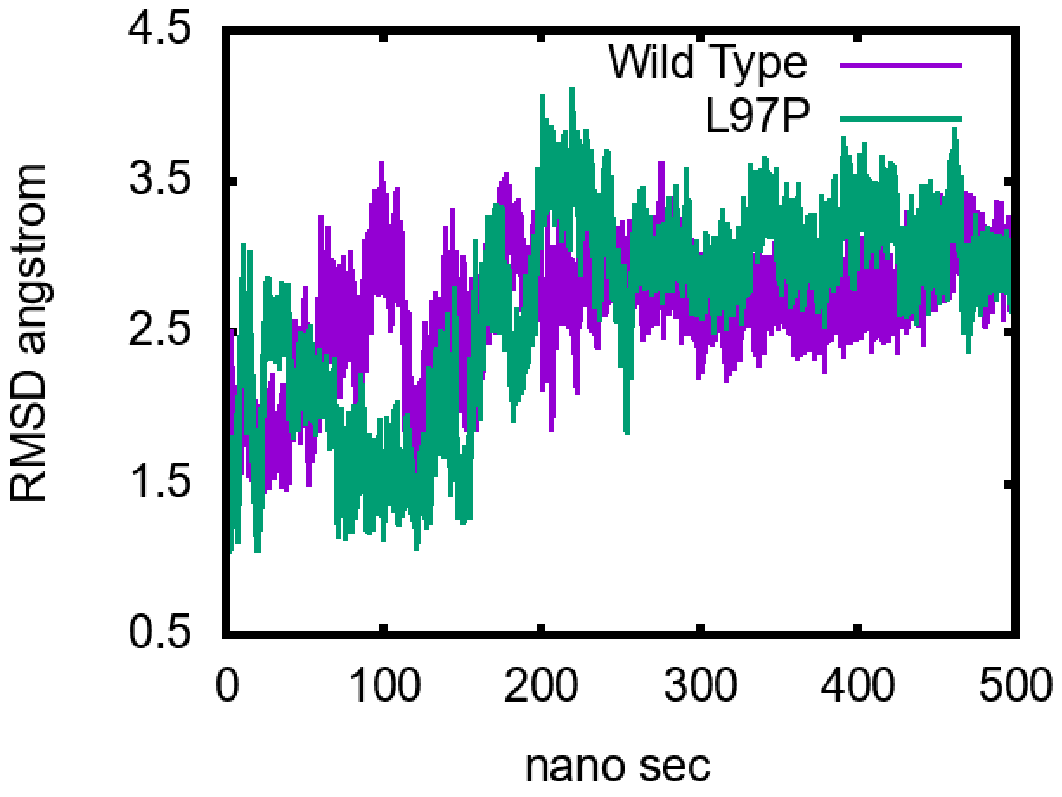

Supplementary Figure 2: Cα RMSD of wild type and L97P UCH-L5 during 500ns MD simulations.

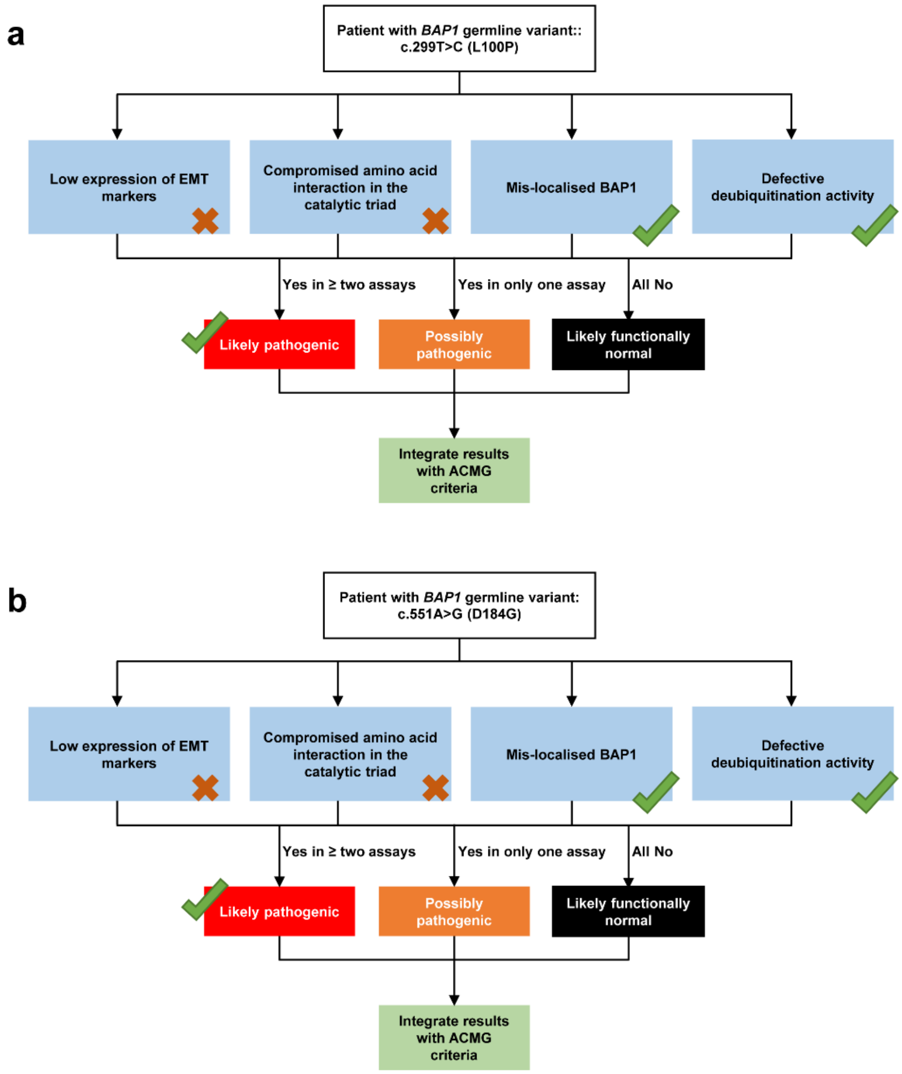

Supplementary Figure 3: *BAP1* VUS c.299T>c and c.551A>G are functionally abnormal. Flowchart to access functionality of *BAP1* VUS c.299T>c (a) and c.551A>G (b).

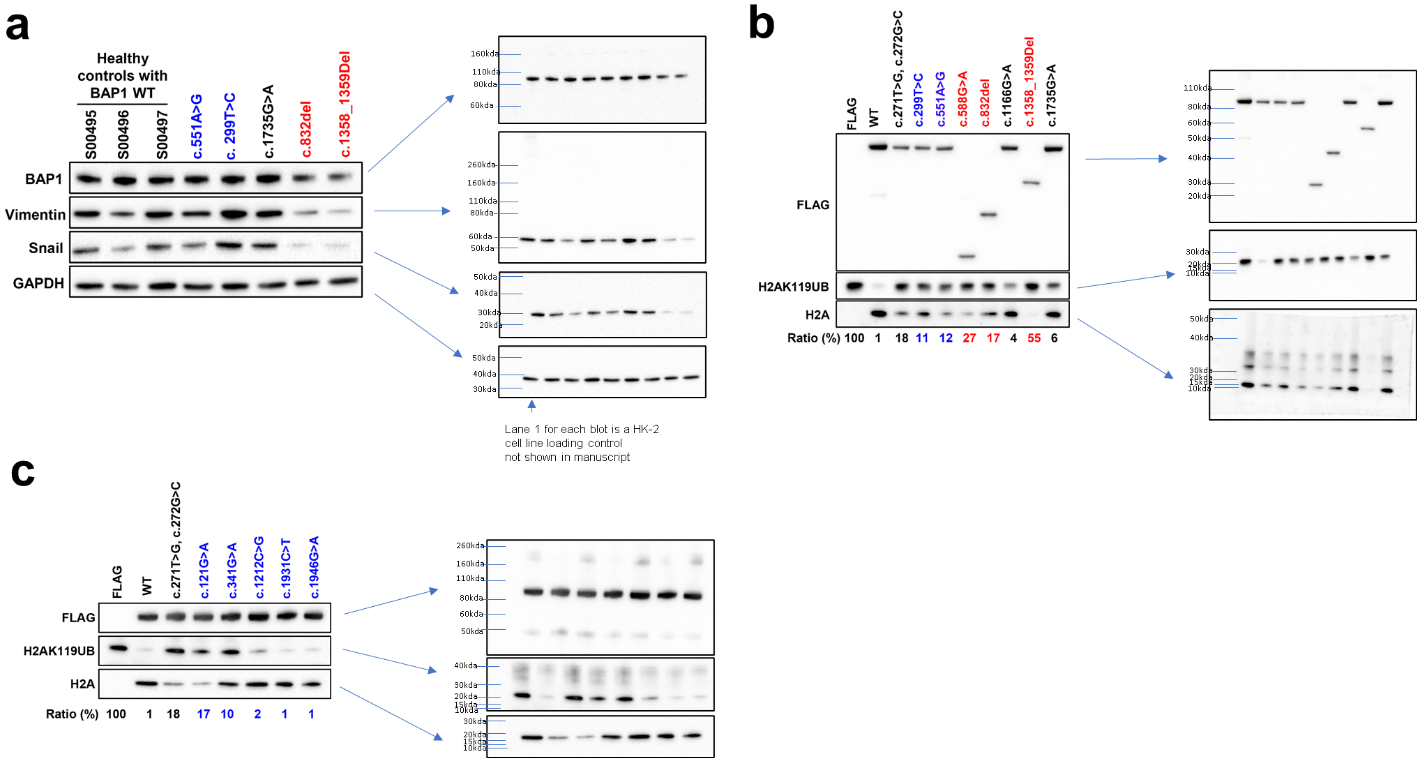

Supplementary Figure 4: Uncropped images of immunoblots. Uncropped images of immunoblots for Figure 1d (a), Figure 4d (b) and Figure 6c (c).
